# Incidental Ascending Colon Ganglioneuroma in the Setting of Hematochezia

**DOI:** 10.7759/cureus.9447

**Published:** 2020-07-28

**Authors:** Christian Nehme, Sami Ghazaleh, Dipen Patel, Syed Hasan, Ali Nawras

**Affiliations:** 1 Internal Medicine, University of Toledo Medical Center, Toledo, USA; 2 Gastroenterology and Hepatology, University of Toledo Medical Center, Toledo, USA

**Keywords:** ganglioneuroma, hematochezia

## Abstract

Ganglioneuromas are slow-growing hamartomatous tumors that are rarely found in the colon. There are three subtypes of ganglioneuromas: polypoid ganglioneuroma, ganglioneuromatous polyposis, and diffuse ganglioneuromatosis. They are differentiated depending on their endoscopic and histological characteristics. Patients with colonic ganglioneuroma may present with nonspecific symptoms; however, they are usually asymptomatic. We present a case of hematochezia, where an ascending colon ganglioneuroma was found incidentally on diagnostic colonoscopy. We will explain how to distinguish the three subtypes of ganglioneuroma on colonoscopy and will mention the genetic disorders associated with them. We will also discuss the treatment of ganglioneuromas.

## Introduction

Ganglioneuromas are slowing-growing tumors that are rarely found in the colon. There are three subtypes of ganglioneuromas: polypoid ganglioneuroma, ganglioneuromatous polyposis, and diffuse ganglioneuromatosis [1]. They are differentiated based on certain endoscopic and histological characteristics. Patients with colonic ganglioneuromas may present with nonspecific symptoms such as abdominal pain, bleeding, or change in bowel habits; however, these patients are usually asymptomatic. We present a case of hematochezia, where an ascending colon ganglioneuroma was found incidentally on diagnostic colonoscopy.

## Case presentation

**Figure 1:**
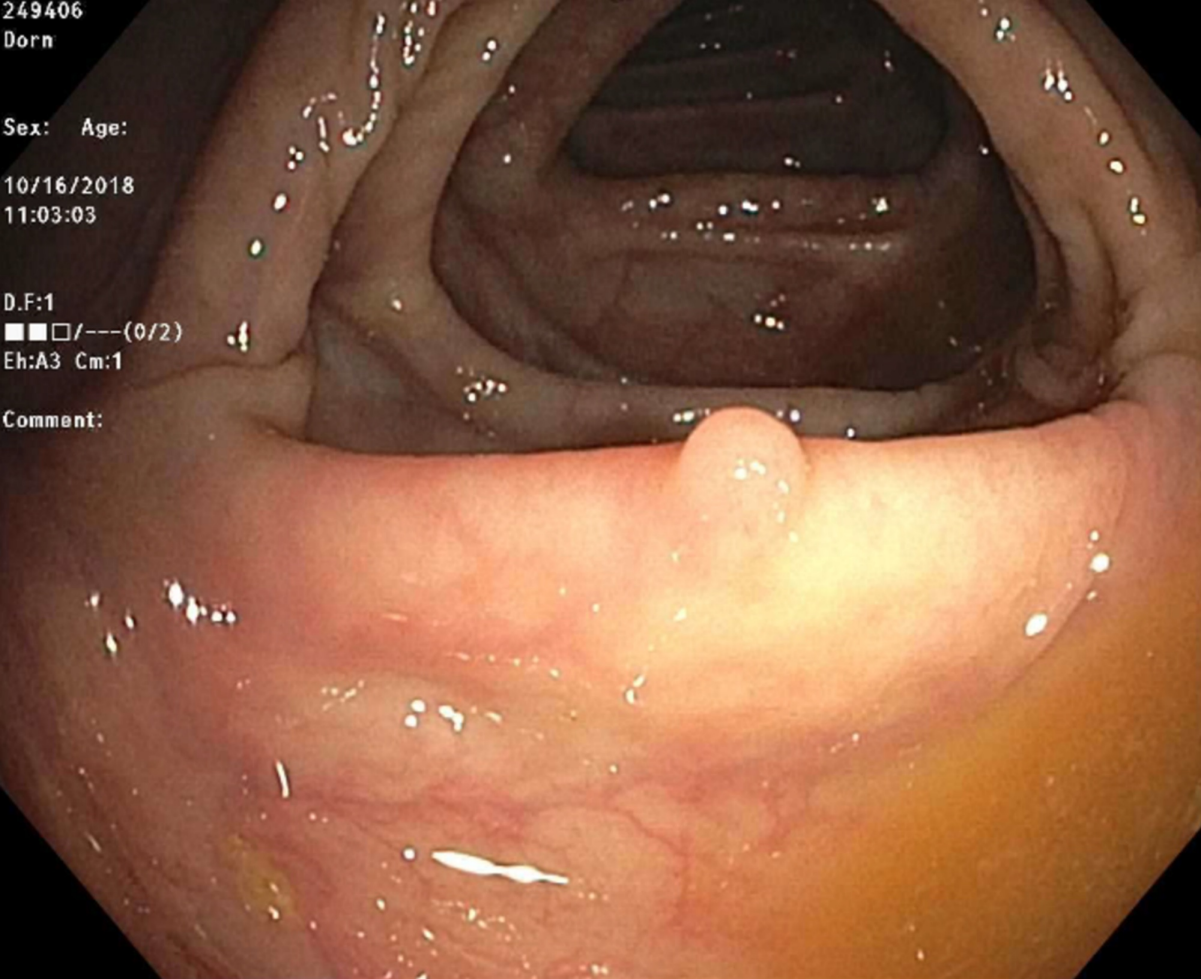
Ascending colonic polyp

**Figure 2:**
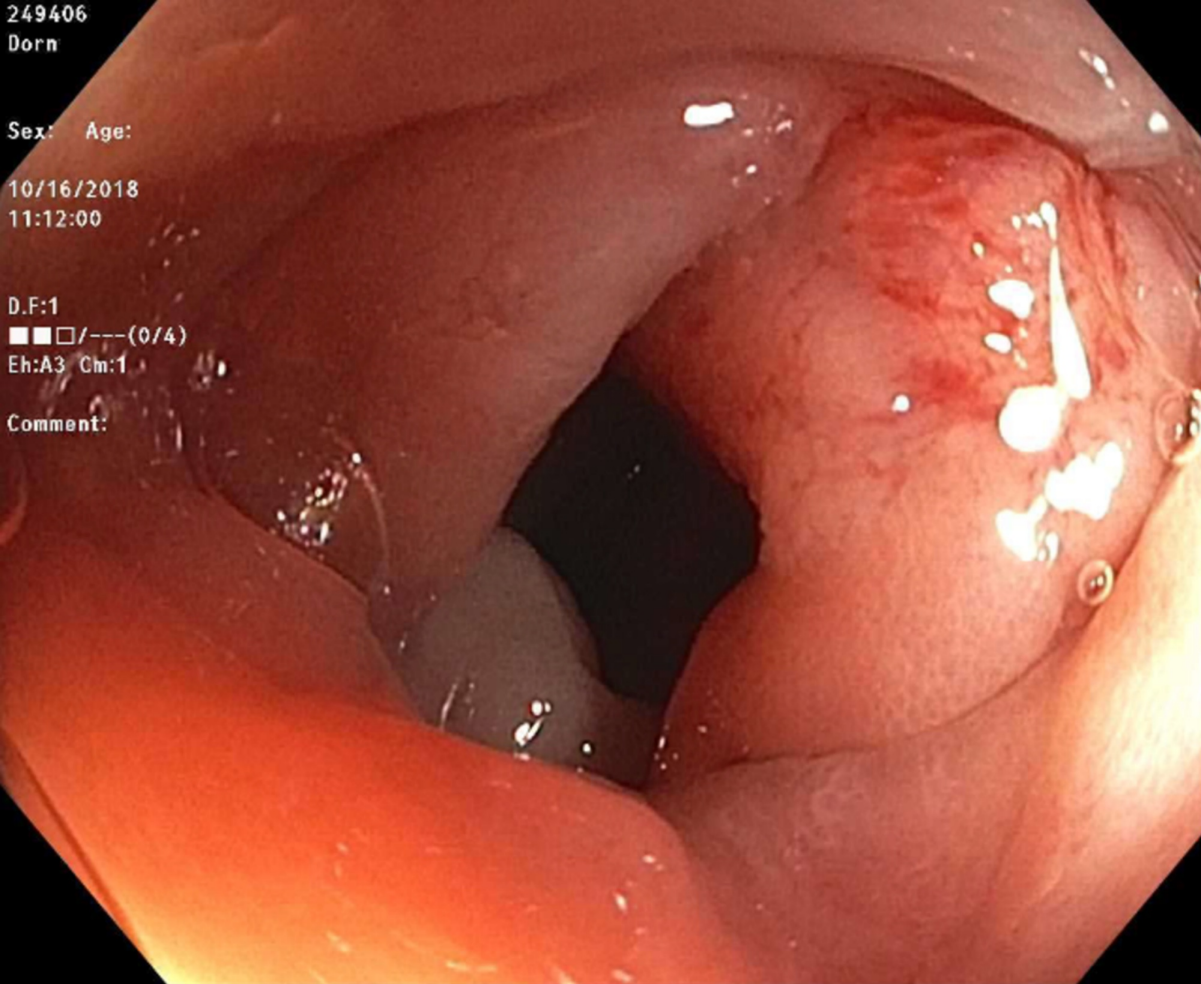
Ascending colon after polypectomy

An 84-year-old male patient with a past medical history of hypertension, chronic atrial fibrillation on rivaroxaban, chronic obstructive pulmonary disease, and prostate cancer status post-radiation presented to the hospital with hematochezia of two-day duration. He had three loose and bloody bowel movements on the day of presentation, with bright red blood and clots present. Hematochezia was associated with nausea and diarrhea. However, the patient denied abdominal pain, vomiting, hematemesis, melena, or weight loss. Last colonoscopy around seven years ago was normal. Vital signs were within the normal limits. Physical examination showed an irregularly irregular rhythm. Abdominal examination was unremarkable. Rectal examination showed dark red stools without any external hemorrhoids. Routine labs were significant for a drop of hemoglobin from 12.5 g/dL to 11 g/dL. International normalized ratio was 1.4. Colonoscopy was performed five days after stopping Xarelto®. It showed a 3-mm polyp in the ascending colon (Figure 1), a 4-mm polyp in the descending colon, and diverticulosis in the descending colon. The 3-mm polyp in the ascending colon was removed with a cold biopsy forceps (Figure 2). Histological examination of the specimen showed ganglioneuroma.

## Discussion

Ganglioneuromas are hamartomatous tumors that usually occur in the head, neck, or adrenal glands. They are rarely seen in the gastrointestinal tract. They originate from undifferentiated neural crest cells, although the exact mechanism of their formation is unknown. It is hypothesized that a soluble nerve growth factor may contribute to their development [2]. There are three different subtypes of ganglioneuromas: polypoid ganglioneuroma, ganglioneuromatous polyposis, and diffuse ganglioneuromatosis [1]. The most common type is polypoid ganglioneuroma, which is usually a small single polyp (or less commonly multiple small polyps) arising from the mucosa and submucosa. These polyps can be sessile or pedunculated. They can also be adenomatous or hyperplastic. The second type of ganglioneuroma is ganglioneuromatous polyposis. This is distinguished from the polypoid subtype by numerous polyps that are similar in size and are histologically identical to polypoid ganglioneuroma. These can also be mucosal, submucosal, sessile, or pedunculated. The third type is diffuse ganglioneuromatosis, which is poorly demarcated and a larger lesion, up to 17 cm in size. It is nodular and consists of diffuse tissue that involves the myenteric plexus [1]. Diffuse ganglioneuromatous can be either mucosal or transmural. Mucosal lesions are more common in adults, whereas both lesions can occur in children.

Patients with colonic ganglioneuromas can be either asymptomatic or present with abdominal pain, hematochezia, constipation, diarrhea, ileus, weight loss, or obstruction. The presence of these symptoms and their severity depend on the size, location, and subtypes of the lesions. On endoscopy, the different subtypes of ganglioneuromas are distinguished based on the number and size of the polyps. Polypoid ganglioneuroma can be either a single lesion or a few lesions less than 2 cm in size. It is not possible to differentiate them from hyperplastic or adenomatous polyps by endoscopy, and, as a result, diagnosis can only be made through histological examination. Ganglioneuromatous polyps are more numerous. They are usually greater than 20, but each polyp is less than 2 cm in size. Diffuse ganglioneuromatous are large, ranging from 1 to 17 cm in diameter. They usually spare the ileum and may distort surrounding tissue. Ganglioneuromas can be associated with genetic disorders depending on their subtype. Single lesions such as polypoid ganglioneuroma are usually an isolated finding. They are not associated with any genetic syndrome. Patients with ganglioneuromatous polyposis can have associated anatomical findings such as several skin tags or cutaneous lipomas that can be present either on the face or on the extremities [1]. Diffuse ganglioneuromatosis may be isolated, but they are typically associated with multiple endocrine neoplasia (MEN) IIb or neurofibromatosis 1 [3,4]. They can also be associated with non-familial adenomatous polyposis, juvenile polyposis, Cowden’s disease, and Ruvalcaba-Myhre-Smith’s syndrome. There are also a few cases where colon cancer, ganglioneuromatous polyposis, or diffuse ganglioneuromatosis are present in the same patient [5]. Due to the high risk of cancer with syndromes associated with ganglioneuromas, patients should be screened for tumors present in the colon, thyroid, breast, and uterus [6]. Screening for MEN IIb can include tests such as serum calcium and calcitonin in addition to urine vanillylmandelic acid. Genetic testing is recommended if there is high suspicion of a genetic disorder.

Treatment of colonic ganglioneuromas depends on their size, location, and subtype, as well as the patient’s clinical history. Treatment also depends on the presence of complications such as bleeding or obstruction. Polypoid ganglioneuroma is a benign lesion; therefore, endoscopic resection using a hot biopsy forceps is usually curative. Although there are not any recommendations on how to further manage polypoid ganglioneuroma, it may be useful to perform a follow-up colonoscopy to ensure complete excision of the lesion [7]. The treatment for large polypoid lesion, ganglioneuromatous polyposis, and diffuse ganglioneuromatosis includes surgical resection of the involved part of the bowel in addition to screening and testing for any associated tumor or genetic syndrome.

Definitive diagnosis of ganglioneuroma can only be made through pathological and histological examination. Histologically, ganglion cells are seen mixed with proliferation of Schwann cells. Hematoxylin and eosin staining will show multiple spindle cells, which is usually enough to confirm the diagnosis. Usually, these lesions are immunoreactive to neuron-specific enolase and S100 protein stain, which is helpful in confirming the neural origin of the legion [8]. There are few case reports of intestinal ganglioneuroma presenting with bleeding [7,9].

## Conclusions

In summary, colonic ganglioneuromas are very rare. We presented a case of a patient who presented to the hospital with hematochezia. An ascending colon polyp, which turned out to be a ganglioneuroma, was found incidentally on colonoscopy. Bleeding may have been caused by the ganglioneuroma or may be unrelated.
